# Popliteal artery entrapment syndrome: a case report with literature review

**DOI:** 10.11604/pamj.2021.39.80.27536

**Published:** 2021-05-27

**Authors:** Taha Abu Al-Tayef, Abdellah Rziki, Hammam Rasras, Omar El Mahi, Adnane Benzirar

**Affiliations:** 1 Department of Vascular Surgery, Mohammed VI University Hospital, Mohammed First University of Oujda, Oujda, Morocco,; 2 Laboratory of Epidemiology, Clinical Research and Public Health, Faculty of Medicine and Pharmacy, Mohammed the First University of Oujda, Oujda, Morocco

**Keywords:** Popliteal artery, claudication, entrapment syndrome, stenosis, case report

## Abstract

Popliteal artery entrapment syndrome generally causes calf claudication in young active adult. It is resulting of the anatomical relationship between the popliteal artery and adjacent muscles or fibrous bands in the popliteal fossa. We present the case of a 36-year-old male with left calf claudication limb in whom popliteal artery entrapment syndrome was diagnosed, and successfully treated surgically.

## Introduction

Popliteal artery entrapment syndrome (PAES) is one of the most common causes of serious disability among young adults and athletes, yet it is underdiagnosed. PAES refers to popliteal artery compression caused by an abnormal anatomy of the nearby musculotendinous structures or surrounding muscle hypertrophy, which can cause various severe complications. We report a case of a young adult patient in whom surgical treatment of PAES was successful.

## Patient and observation

A 36-year-old male with no specific medical history, who was a runner and weight lifter for 10 years, presented with left leg intermittent claudication that appeared in the last 7 months. The pain limited his walking perimeter to 200 meters and was relieved by short rest. It also occurred while driving a car for more than one hour. Physical examination revealed a pulse rate of 65/min and a blood-pressure of 120/80 mmHg. No cardiac abnormality was detected. All pulses were palpable in the lower right limb and only the femoral pulse was palpable in the left one, with an ankle to brachial index (ABI) of 0.4 compared to a normal ABI in the right limb. The left popliteal pulse could be found by using the pocket vascular Doppler, it disappeared with the dorsiflexion of the foot.

**Figure 1:**
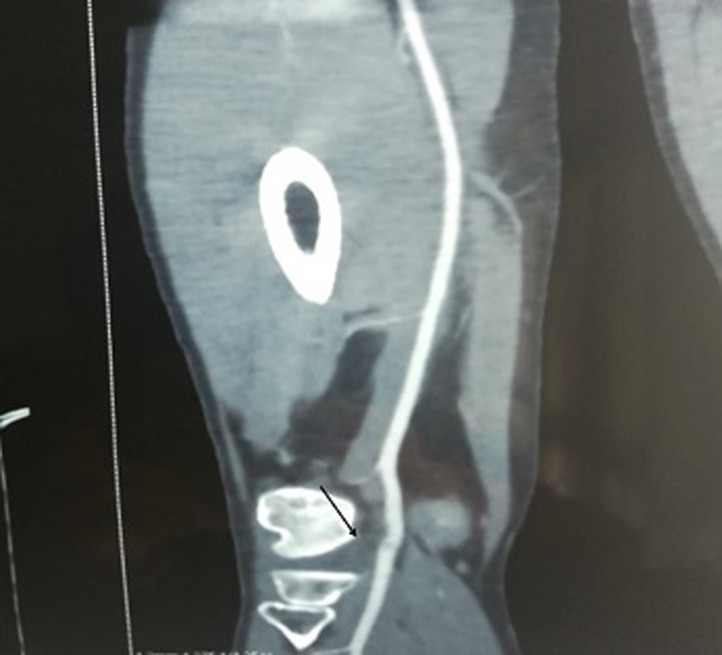
computerized tomography (CT) angiography showing the stenotic segment of the right popliteal artery (arrow)

**Figure 2:**
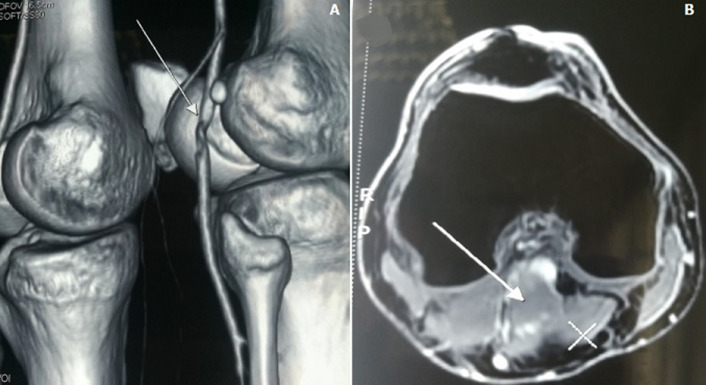
magnetic resonance angiography (MRA) of the popliteal fossa showing (A) the stenotic segment of the popliteal artery (arrow), notice the decreased blood flow downstream; (B) the abnormal musculotendinous insertion of the medial gastrocnemius head (arrow)

Doppler ultrasound showed a monophasic reduced systolic blood flow in the popliteal and posterior tibial arteries, the blood flow velocity was lower in the distal vessels. Computerized tomography (CT) - angiography of the popliteal fossa in 4-mm sections showed a popliteal artery stenosis with a post-stenotic dilatation (Figure 1). A magnetic resonance angiography (MRA) showed the entrapment of the popliteal artery by an accessory slip of the medial head of the gastrocnemius muscle. We note that an atheroma plaque of 14 mm was also found reducing partially the popliteal artery lumen with a low flow downstream (Figure 2).

**Figure 3:**
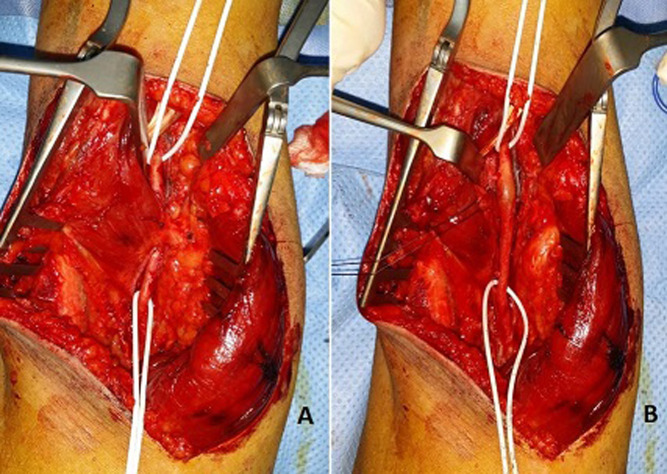
operative photos showing (A) the anomalous accessory slip of the medial head of the gastrocnemius muscle (arrow) and (B) the post-stenotic popliteal aneurysm after resection of the anomalous muscle

**Figure 4:**
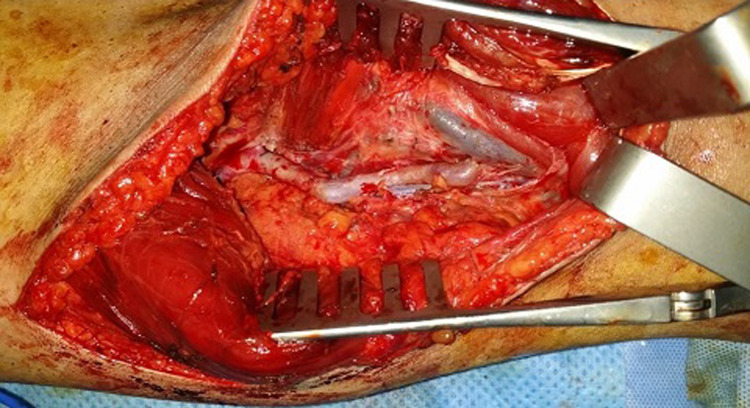
final replacement of the narrowed popliteal segment by a saphenous venous graft bypass

**Figure 5:**
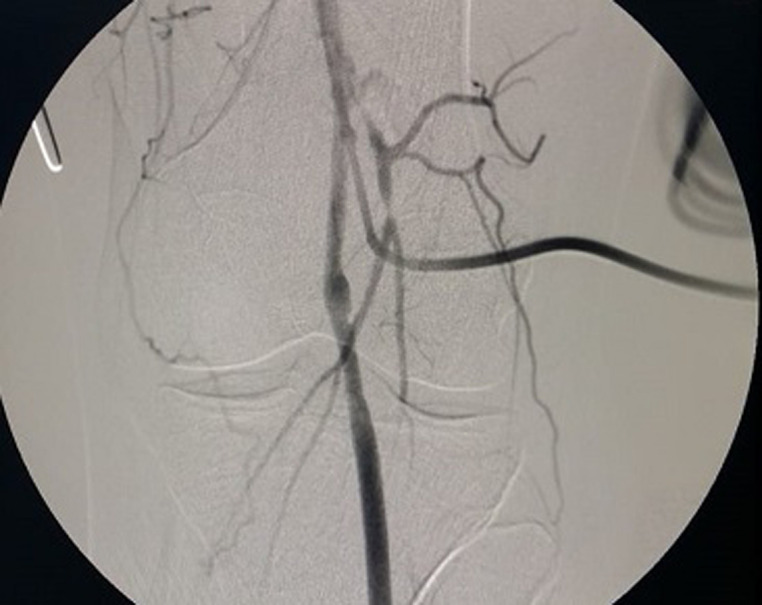
post-operative angiography objectifying the relieve of the popliteal stenosis

Surgical repair was decided using a posterior approach of the popliteal artery. An abnormal insertion into the medial femoral condyle of the accessory head of the gastrocnemius muscle was found (Figure 3). The popliteal artery was angulated and trapped behind the muscle with a post-stenotic dilatation. An autologous saphenous venous graft was used to replace the compromised arterial segment, and the accessory head the gastrocnemius muscle was resected (Figure 4). An angiographic control (Figure 5) allowed confirmation of the stenosis relief and the restitution of a good blood flow. The patient had an uneventful postoperative course and was discharged home on the 8^th^ postoperative day.

## Discussion

Anderson Stuart was the first to describe the anatomical basis of popliteal entrapment in 1879 [1]. Love and Whelan, of Walter Reed General Hospital in the United States, introduced the term “popliteal artery entrapment syndrome” (PAES) in 1965 [2]. PAES prevalence ranges between 0.16-3.5% in general [3]. It is responsible of 40% of cases of intermittent claudication in young patients who usually don´t have risk factors of atherosclerosis and who are healthier and more active than average for their age group, which makes appropriate diagnosis difficult [4-6]. PAES occurs due to an extrinsic compression of the popliteal vessels that results in vascular damage, which can lead to aneurysm, thromboembolism and limb ischemia [7]. PAES can be congenital, caused by abnormal embryological development of the popliteal artery or of the myofascial structures in the popliteal fossa [8], or acquired, which has been reported in high-performance athletes, such as cyclists [9].

The most used classification of PAES is the Rich´s classification into five types based on the embryologic aspect [8, 10]: in type I PAES, the popliteal artery has an internal deviation and is displaced medial to and beneath the gastrocnemius muscle or its tendon. In type II PAES, the popliteal artery is compressed due to an abnormal lateral insertion of the medial gastrocnemius muscle. In type III PAES, popliteal artery is compressed by an additional tendon of the gastrocnemius muscle that inserts laterally. In type IV PAES, both popliteal artery and vein are compressed. Type V PAES is the functional PAES where popliteal vessels have normal anatomy but are compressed due to muscular hypertrophy. Our patient had a type III entrapment due to compression of the popliteal artery by the accessory slip of the medial head of the gastrocnemius muscle, which is the most common type with a 35% prevalence rate [5]. PAES can be diagnosed with a combination of clinical presentation and findings, and imaging studies including computed tomography, magnetic resonance imaging and Doppler sonography [11].

Physical examination may find decreased or absent pulses during forced dorsal foot flexion, or signs of decreased perfusion such as pallor, poikilothermia and limb cyanosis in acute cases [12]. Ultrasonography can be useful to confirm stenosis or occlusion of popliteal vessels, but it is technically difficult due to the constant motion of the structures during muscle contraction [13]. Computed tomography can show artery occlusion, but it is not always able to provide detailed information regarding the relationship between vascular and musculotendinous structures within the popliteal fossa, unlike magnetic resonance imaging that has been shown to be the best method of evaluation of the anatomy of the popliteal fossa [14,15]. Williams C *et al*. [13] published a new diagnosis approach to PAES combining ultrasonography and magnetic resonance imaging techniques with dynamic plantarflexion of the ankle against resistance, which allows the diagnosis of anatomical and functional entrapment types and management guidance either with surgery or Botox injection [9]. Angiography can provide interesting information to plan intervention. If no changes are seen on vascular imaging in patients with a high degree of diagnostic suspicion for PAES, the patient may be asked to perform plantar dorsiflexion until becoming symptomatic, then angiography can be repeated [15].

For patients with anatomical PAES, surgery is indicated even when the patient is asymptomatic to avoid irreversible damage [5], it consists in decompression of the popliteal artery, with arterial reconstruction in case of degeneration or occlusion of the popliteal artery [6]. Posterior approach of the popliteal fossa allows better visualization of the popliteal structures, medial access is more commonly used in cases of large popliteal occlusion requiring femoropopliteal bypass [16]. Arterial bypass using venous graft has been reported to be superior to arterioplasty using venous patch [17]. Treatment of the popliteal artery stenosis by angioplasty can be considered only after removing of the underlying reason of arterial entrapment, otherwise the treatment won´t be effective with a high risk of restenosis [3]. More than 90% of patients have significant improvement in their symptoms and return to normal physical activity within three months after surgery [18]. However, surgical outcome for patients with functional PAES have been reported to be worse when compared with anatomical PAES [19]. Intramuscular injection of Botox to induce paralysis of the compressing gastrocnemius muscle have been reported, although clinical controlled studies are needed to support the efficacy of this procedure for patients with functional PAES [9].

## Conclusion

Popliteal artery entrapment syndrome is a underestimated etiology of claudication in young sportive people. RMA is the main tool for making its diagnosis. Prognosis depends on the delay of treatment, as any delay can threaten the functional prognosis. Surgical treatment is the treatment of choice.
